# Effect of Lifestyle Intervention in the Concentration of Adipoquines and Branched Chain Amino Acids in Subjects with High Risk of Developing Type 2 Diabetes: Feel4Diabetes Study

**DOI:** 10.3390/cells9030693

**Published:** 2020-03-12

**Authors:** Itziar Lamiquiz-Moneo, Ana M. Bea, Cristian Palacios-Pérez, Pilar De Miguel-Etayo, Esther M. González-Gil, Chuan López-Ariño, Fernando Civeira, Luis A. Moreno, Rocio Mateo-Gallego

**Affiliations:** 1 Hospital Universitario Miguel Servet, Instituto de Investigación Sanitaria Aragón (IIS Aragón), CIBERCV, Universidad de Zaragoza, 50009 Zaragoza, Spain; anamariabeasanz@hotmail.com (A.M.B.); 724630@unizar.es (C.P.-P.); 740925@unizar.es (C.L.-A.); civeira@unizar.es (F.C.); rmateo@unizar.es (R.M.-G.); 2 Growth, Exercise, Nutrition and Development (GENUD) Research Group, Instituto Agroalimentario de Aragón (IA2), Universidad de Zaragoza, Instituto de Investigación Sanitaria de Aragón (IIS Aragón), 50009 Zaragoza, Spain; pilardm@unizar.es (P.D.M.-E.); esthergg@unizar.es (E.M.G.-G.); lmoreno@unizar.es (L.A.M.); 3 Centro de Investigación Biomédica em Red de Fisiopatología de la Obesidad y la Nutrición (CIBEROBN), Instituto de Salud Carlos III, 28020 Madrid, Spain; 4 Department of Biochemistry and Molecular Biology II, Instituto de Nutrición y Tecnología de los Alimentos, Center of Biomedical Research (CIBM), Universidad de Granada, 18071 Granada, Spain; 5 Departamento de Medicina, Psiquiatria y Dermatologia, Facultad de Medicina, Universidad de Zaragoza, 50009 Zaragoza, Spain; 6 Departamento de Fisiatría y Enfermería, Facultad de Ciencias de la Salud y del Deporte, Universidad de Zaragoza, 22002 Huesca, Spain

**Keywords:** Feel4Diabetes study, branched chain amino acids, retinol-binding protein 4, type 2, diabetes lifestyle intervention

## Abstract

Introduction: The global prevalence of type 2 diabetes (T2D) is increasing rapidly, especially in low- and middle-income countries and has a high number of associated comorbidities. Plasmatic concentrations of branched chain amino acids (BCAA) and retinol-binding protein 4 (RBP4) have been shown to be elevated in T2D subjects in cross-sectional studies. However, the effect of lifestyle community-based interventions on BCAA and RBP4 concentrations has not yet been analyzed. Material and methods: The Feel4Diabetes study is a school and community-based intervention that identified 360 European families with a high risk of developing T2D according to the FINDRISC questionnaire. Families were randomized in control and intervention groups were followed-up from 2016 to 2018. In the Spanish families, the concentration of BCAA and RBP4 was determined in 266 subjects (115 control and 151 intervention group) that attended the three time-point assessments by colorimetric and ELISA reaction, respectively. Results: Baseline BCAA levels showed positive correlations with the FINDRISC score and glucose impairment (baseline glucose, insulin, and glycated hemoglobin), body mass index, and body weight. The participants receiving the community-based intervention showed a significant decrease in glycated hemoglobin and BCAA levels compared to the control group (*p* = 0.011 and *p* < 0.001, respectively). However, baseline RBP4 did not show significant correlations with anthropometric and glycemic parameters, and no significant change was observed in anthropometric parameters and RBP4 concentrations throughout the follow-up. Conclusion: A community-based intervention on lifestyle led to a significant reduction in BCAA levels regardless of weight loss. These findings suggest that this interventional approach could be promising in T2D prevention.

## 1. Introduction

Type 2 diabetes (T2D) doubles the risk of cardiovascular disease mortality and it is associated with increased morbimortality risk from numerous other causes and reduced quality of life [1,2]. The global prevalence of T2D is increasing rapidly, especially in low- and middle-income countries, which is mainly attributable to environmental and behavioral risk factors [3,4]. Finding lifestyle intervention strategies that are effective at preventing or delaying the onset of T2D and early identification of those individuals at high risk constitute a priority for the scientific community [5].

Metabolomics have recently found biochemical changes that occur before T2D onset and can elucidate its pathophysiology to facilitate new approaches to prevention and management [6,7].

Branched-chain amino acids (BCAA) have been identified as potential contributors to glucose impairment, insulin resistance, and increased T2D incidence risk, although the responsible mechanisms for these effects are not fully understood [8,9,10]. It has been demonstrated that intracellular accumulation of BCAA activates the mammalian target of rapamycin (mTOR), resulting in insulin signaling inhibition [10,11]. Recently, brown adipose tissue has been founded to have an essential role in BCAA clearance [12]. Thus, impaired brown adipose tissue activity in conditions such as obesity or T2D decreases systemic BCAA clearance. Adipose tissue dysfunction is characterized by hypertrophy, increased autophagy and apoptosis, and inflammatory processes, among others [13]. Adipocytes release adipokines, whose circulating levels reflect adipose tissue impairment and are directly linked to cardiometabolic diseases. Retinol-binding protein 4 (RBP4) is a recently recognized adipokine, and several epidemiological studies have demonstrated that elevated serum RBP4 concentrations play a critical role in the development of insulin resistance and T2D [14,15]. Among adipokines, RBP4 is the one most related to glycemic homeostasis and other cardiometabolic parameters (like triglycerides) regardless of weight loss. However, the effect of lifestyle interventions in studies with a large sample size has barely been explored. Besides, other previous studies have demonstrated that RBP4 levels seem to be quite modifiable by style–life modification, decreasing with hyper protein hypocaloric diets and directly associated with triglyceride concentration improvement [16]. Most of the studies exploring the association between BCAA and RBP4 with glucose metabolism impairment are observational and they have been carried out in subjects with prediabetes or T2D. However, while subjects with high risk of developing T2D have elevated levels of BCAA and RBP4; whether they are related to glucose homeostasis and lifestyle intervention which could impact their concentrations has not been previously explored and could be useful to find early markers of disease onset. Thus, the aim of this study is to explore the circulating levels of BCAA and RBP4 in adult subjects with high risk of developing T2D, based on a validated questionnaire (Finnish Diabetes Risk Score, FINDRISC), to find if they are related to glucose parameters and if a community-based lifestyle intervention has a significant impact on their concentrations.

## 2. Material and Methods

### 2.1. Study Population and Design

The current study has been developed to include Spanish participant data from the Feel4Diabetes-study, which is an EU-funded study aimed at carrying out, implementing, and evaluating school- and community-based interventions to prevent T2D in families from vulnerable population groups across Europe. The Feel4Diabetes-study is registered at clinicaltrials.gov as NCT02393872, and further information about it can be found at the following website: http://feel4diabetes-study.eu/.

The methodology of the study has been previously explained in depth [17]. Briefly, the study recruited participants from vulnerable population-groups in six European countries, including Bulgaria, Hungary, Belgium, Finland, Greece, and Spain. Those families within schools from municipalities with the lowest educational levels or the highest unemployment rates were considered as vulnerable groups and were recruited in the city of Zaragoza (Spain). Participants at high risk of developing T2D were identified through the FINDRISC questionnaire [17]. Previous studies have demonstrated that the FINDRISC questionnaire has good validity for identifying subjects at increased risk of T2D and is the most used diabetes risk score in Europe [18,19,20]. In this study, families were contacted at schools and those participants with a FINDRISC score ≥9 were considered as high risk. Families with at least one member at high risk based on FINDRISC were considered as “high risk families”. The randomization to the intervention and control groups was performed at a municipality level (1:1 ratio) after baseline measurements were completed. Thus, the schools and the families (both all families and “high risk families”) within each municipality were automatically allocated to the intervention or control group. “High risk” participants had follow-ups at the Hospital Universitario Miguel Servet where clinical, anthropometric, and biochemical parameters were collected at baseline (2016), after one year of the follow-up (Follow-up 1, 2017) and after two years of the follow-up (Follow-up 2, 2018).

Lifestyle intervention was implemented from 2016 to 2018, which has already been explained in detail elsewhere [17]. It has two different components: (1) the “all families” component, which was developed at schools, home, and the local municipalities; (2) “high risk families”, which was delivered out of the school setting for just these families. For example, the “all families” component involved activities at school aiming to increase water consumption (instead of sugary drinks) or to increase the intake of fruits and vegetables, among others. Furthermore, changes in infrastructure and human resources at the municipality level were promoted to facilitate healthy lifestyle adherence and behavioral changes of families. The “high risk families” component included seven lifestyle counseling sessions spread over the academic years 2016–2017 that were focused on (a) decreasing body weight by 5% (if there were overweight or obese participants); (b) increasing the consumption of whole grains, nuts, low-fat dairy, and olive and rapeseed oils; and (c) decreasing the intake of snacks, fast foods, red and processed meat. The seventh session consisted of an individual visit of the high-risk participant who underwent the clinical visit by including medical check-up discussions and setting specific and feasible goals within every lifestyle change. High risk participants in the control group also received this personal session, which consisted of a medical check-up explanation and general healthy lifestyle counseling. “High risk families” received motivational messages on their mobile phones during the second year of the study (2017) with the objective of reaching the healthy lifestyle goals previously stated.

### 2.2. Anthropometric, Clinical, and Biochemical Parameters

These parameters were collected in high risk participants both in the control and intervention groups at baseline, Follow-up 1, and Follow-up 2. Bodyweight was measured twice without shoes with a calibrated scale (Seca 813, Seca Deutschland®, Hamburg, Deutschland, Germany). If the difference between the two measurements was greater than 0.1 kg, a third assessment was performed. Height was assessed twice with a wall-mounted stadiometer (Seca 217, Seca Deutschland®, Hamburg, Deutschland, Germany). If the difference between the two measurements was greater than 0.1 cm, a third assessment was carried out. BMI was calculated as weight in kilograms divided by the square of height in meters. Blood pressure was measured in triplicate with a validated semiautomatic oscillometer (Omron M3, Omron Cop; Hoofdorp, the Netherlands). All measurements were taken in accordance with the recommended guidelines: no food or drink 3 h prior to measurements, no exhausting exercise 12 h prior to measurements, and no alcohol or caffeine consumption 24 h prior to measurements. We calculated the mean of two or three measurements, if applicable, for these parameters.

Blood samples were drawn by venipuncture after at least 10 hours of overnight fasting. The levels of total cholesterol (TC), triglycerides (TG), and HDL cholesterol (HDLc), uric acid, gamma-glutamyl transferase (GGT), glutamic-pyruvic transaminase (GPT), and aspartate transaminase (AST) were determined by standard enzymatic methods. LDL cholesterol levels were calculated with the Friedewald’s formula when serum TG was <400 mg/dL. Non-HDLc was calculated as total cholesterol minus HDLc. Blood glucose concentration was measured with the glucose-oxidase method. Insulin levels were measured via radioimmunoassay and homeostasis model assessment of insulin resistance (HOMA–IR) and were estimated as fasting serum glucose (mg/dL) × plasma insulin (μU/mL)/405. Glycated hemoglobin (HbA1c) was determined via high-performance liquid chromatography.

### 2.3. Dietary and Physical Parameters

To evaluate the impact of the Feel4Diabetes-intervention, the participants completed standardized questionnaires, including information about drinking, eating, physical activity, and sedentary habits. Based on the frequency and quantity of food consumption, we calculated the intake of each food group per week. Physical activity was monitored by pedometers or accelerometers [17].

### 2.4. Branched Amino Acid and Retinol-Binding Protein 4 Quantification

Serum samples were stored at −80 °C until assayed. Serum BCAA (isoleucine, leucine, and valine) were quantified using a branched amino acid kit (MAK003, Sigma-Aldrich, Merck Life Science S.L.U, Madrid, Spain). Serum RBP4 was quantified with the RayBio® Human RBP-4 ELISA kit (RayBiotech, Inc, Peachtree Corners, GA, USA). Both analyses were performed in duplicates, and serum sample dilutions were done according to the detection range of each. Briefly, serum BCAA was determined by adding 13 μL of serum sample with 37 BCAA assay buffer and 50 μL of assay reaction. Leucine standard was prepared from 10 μL of leucine 10 mM with 90 μL of BCAA assay buffer. All samples were incubated for 30 min at room temperature protected from light, immediately the absorbance was measured at 450 nm on SYNERGY HT (BioTek, Instruments, Inc, Winooski, VT, USA) and data was collected and analyzed using GEN5^TM^ software (BioTek, Instruments, Inc, Winooski, VT, USA). RBP4 serum was determined by adding 100 μL of RBP4 standard or dilution samples in the plate incubating the samples for 2 hours and 30 min at room temperature in an orbital plate shaker. Dilution samples and RPB4 standard were decanted, and 100 μL of diluted antibody was added in each well, incubating the mix for one hour at room temperature in an orbital plate shaker. After that, the mix was decanted and 100 μL of diluted streptavidin was added in each well, incubating the mix for 45 min at room temperature in an orbital plate shaker. Then, after decanting the mix again, 100 μL of TMB-One Step was added, incubating the mix for 30 min at room temperature in an orbital plate shaker protected from light. Finally, 50 μL of STOP solution was added to each well; immediately, the absorbance was measured at 450 nm on a SYNERGY HT (BioTek, Instruments, Inc, Winooski, VT, USA) and data was collected and analyzed using GEN5^TM^ software (BioTek, Instruments, Inc, Winooski, VT, USA). Intra and inter-assay coefficients of variation of ELISA assays were less than 10% and less than 12%, respectively.

### 2.5. Statistical Analysis

Continuous variables are expressed as mean ± SD or median (25th–75th percentile), according to their distribution. Categorical variables are reported as *n* (percentages). ANOVA and Kruskal–Wallis tests were performed for the comparison of multiple independent variables. Categorical variables were compared using the chi-squared test by including inter-group comparison. Pearson’s tests of correlation were applied as appropriate. Differences in paired variables were calculated with the dependent *t*-test for paired samples or with the Wilcoxon test, as applicable. To assess differences in RBP4 and BCAA concentrations among groups, the effect of the lifestyle intervention on different cardiometabolic parameter changes and the impact of RBP4 and BCAA in this association, we applied multiple linear regression while including weight loss and sex as independent variables. All statistical analyses were performed with R software (version 3.5.0) and significance was set at *p* < 0.05 [21].

## 3. Results

### 3.1. Baseline and Anthropometric Characteristics

Out of 487 participants who attended the first year (2016), 266 subjects attended the three study visits, and they were included in the current study. Of the 266 subjects, 115 (43.2%) belonged to the control group, while 151 (56.8%) belonged to the intervention group (Figure S1). As shown in Table 1, those participants allocated to the intervention group had significantly higher levels of baseline insulin and HOMA–IR than those in the control group (*p* = 0.027 and *p* = 0.045, respectively). The participants randomized to either the intervention or control groups did not differ in any other clinical, anthropometric, or biochemical characteristics at baseline. Importantly, there were no significant differences in baseline concentrations of BCAA and RBP4 between both groups (*p* = 0.745 and *p* = 0.327, respectively; Table 1).

Table 2 shows baseline clinical, anthropometric, and biochemical characteristics and baseline BCAA and RBP4 concentrations of participants based on the presence of impaired glycemic metabolism. There was a higher prevalence of men in diabetic and prediabetic groups compared to normoglycemic groups (*p* = 0.004). Diabetic and prediabetic subjects had significantly higher baseline body weight, BMI, and TG concentration than control groups, which was especially remarkable in diabetic subjects. We observed expected differences among groups in glycemic metabolism (*p* = 0.043, *p* = 0.045 and *p* = 0.020, respectively). Interestingly, BCAA showed a direct association with impaired glycemic metabolism by founding significantly higher concentration in prediabetic and especially in diabetic subjects than in the control group (*p* = 0.018). However, baseline concentrations of RBP4 have not shown any relationship with impaired glycemic metabolism (*p* = 0.192). Besides, we found that there was not a significant difference between BCAA or RBP4 levels according to diabetic therapy (oral antidiabetic vs. insulin therapy).

### 3.2. Relationship between BCAA and RBP4 with Clinical, Anthropometrics, Glucose Metabolism, Lifestyle and T2D Risk at Baseline

**Figure 1:**
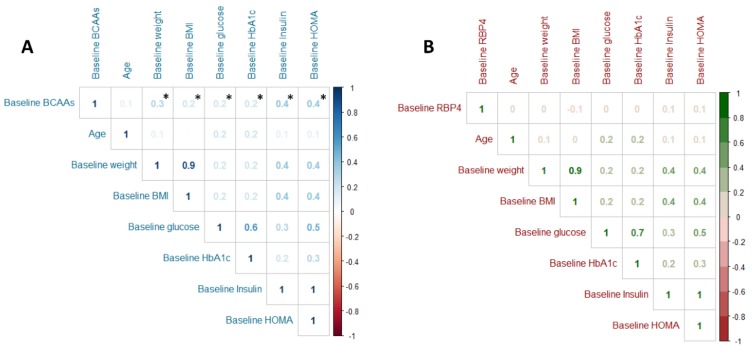
Correlation between baseline levels of BCAA (**A**) or RBP4 (**B**) with anthropometric and diabetes markers (N = 266). BCAAs: branched amino acids; BMI: body mass index; HbA1c: glycated hemoglobin; HOMA: homeostatic model assessment. Correlation analyzed was performed by Pearson’s tests. * Denotes significant difference with *p* < 0.05.

**Figure 2:**
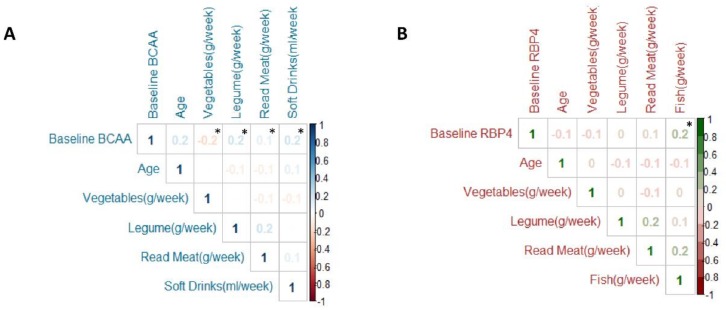
Correlation between baseline dietary items and baseline BCAA (**A**) or RBP4 (**B**) levels (N = 266). BCAA: branched amino acid. Correlation analyzed was performed by Pearson’s tests. * Denotes significant difference with *p* < 0.05.

Baseline BCAA displayed a significant correlation with sex, showing BCAA concentrations were significantly higher in men than women and this difference was maintained throughout the follow-up regardless of whether they belonged to the control or intervention group (*p* < 0.001, Figure S2). Baseline BCAA levels showed a significant positive correlation with anthropometric parameters like baseline body weight and BMI (r = 0.30, *p* < 0.001 and r = 0.20, *p* < 0.001 respectively), and a positive correlation with glycemic markers like glucose, Hb1Ac, insulin, and HOMA-IR (r = 0.20, *p* < 0.001; r = 0.20, *p* < 0.001; r = 0.40, *p* < 0.001; and r = 0.40, *p* < 0.001, respectively; Figure 1A). However, baseline RBP4 concentrations have not showed any correlation with anthropometrics and glucose metabolism parameters (Figure 1B). Baseline BCAA levels had a significant positive correlation with legumes, red meat, and soft drinks consumption (r = 0.20, *p* < 0.001; r = 0.10, *p* = 0.019; and r = 0.20, *p* = 0.009 respectively) and a significant negative correlation to vegetable intake (r = −0.20, *p* = 0.006; Figure 2A). Nevertheless, baseline RBP4 concentrations did not show any correlation with legumes, red meat, soft drinks, and vegetables consumption, although its concentration had a significant and positive correlation to fish consumption (r = 0.20, *p* < 0.001; Figure 2B). Baseline BCAA showed significant positive correlation with baseline gamma glutamyl transferase, transaminase glutamic pyruvic, and systolic blood pressure (r = 0.20, *p* < 0.001; r = 0.20, *p* = 0.002; r = 0.20, *p* < 0.001, respectively) and significant negative correlation with HDLc (r = 0.20, *p* < 0.001, Figure S3A). Baseline RBP4 showed significant positive correlation with baseline TG and diastolic blood pressure (r = 0.20, *p* = 0.013; r = 0.20, *p* < 0.001, respectively; Figure S3B).

**Figure 3:**
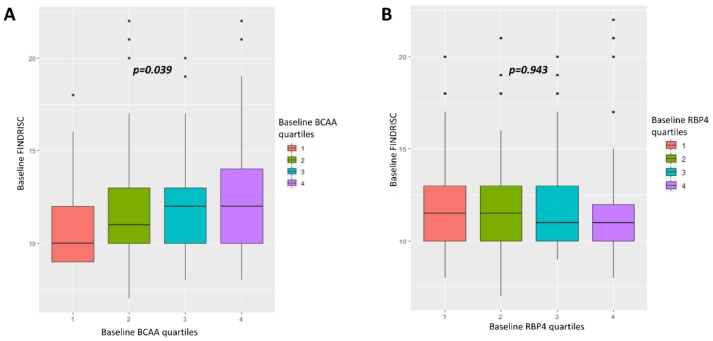
Association between baseline FINDRISC and baseline BCAA and RBP4 levels. BCAA: branched amino acid. RBP4: retinol-binding protein 4. The *p*-value, which compares baseline FINDRISC according to baseline BCAA quartiles (**A**) or baseline RBP4 quartiles (**B**), was calculated by ANOVA test.

Finally, we analyzed the relationship between baseline BCAA and RBP4 levels and the FINDRISC score, which assessed T2D risk. Baseline FINDRISC score showed significant association with baseline BCAA quartiles, indicating that participants with higher values of FINDRISC score were also those with higher baseline BCAA concentrations (*p* = 0.039, Figure 3). However, baseline FINDRISC scores did not show any relation with baseline RBP4 concentrations (*p* = 0.943, Figure 3). When we analyzed the FINDRISC score, according to the items questionnaire, only FINDRISC item–BMI (kg/m^2^), which indicates the self-reported BMI by the participant, had significant association with baseline BCAA levels, showing that participants with higher BMI had significantly higher concentrations of BCAA at baseline (*p* ≤ 0.001, Table S1). Regarding RBP4 levels, only FINDRISC item–blood pressure medication showed a significant relationship. Those participants who reported taking blood pressure medication had significantly higher levels of RBP4 than subjects who did not report taking blood pressure medication (*p* < 0.001, Table S1).

### 3.3. Clinical, Anthropometric, Biochemical, and Lifestyle Parameters across the Study

The evolution of biochemical and anthropometric characteristics of all Spanish subjects who completed the Feel4Diabetes study are shown in Table 3, according to randomized grouping. Both control and intervention groups showed a significant decrease in body weight in Follow-up 1, but no significant decrease in Follow-up 2 compared to the baseline, with no difference between groups. Only the intervention group showed a statistically significant decrease in BMI after one year of study compared to baseline. Regarding lipid profiles, the control group showed a significant decrease in TG and HDLc levels during the first year of the study. Besides, the TG levels also continued to decrease during the second year in the control group. The intervention group did not show any significant decline of TG, but they showed a significant increase in HDLc concentrations, which could be explained by the rise of physical activity in this group. Concerning glycemic parameters, only the control group had a significant increase in their glucose levels at Follow-up 1, while the intervention group did not have a significant change in their glucose concentration. Interestingly, HbA1c significantly dropped in both groups, although the decrease in the intervention group was significantly higher than in the control group throughout the follow-up (*p* = 0.011, comparing both groups).

**Figure 4:**
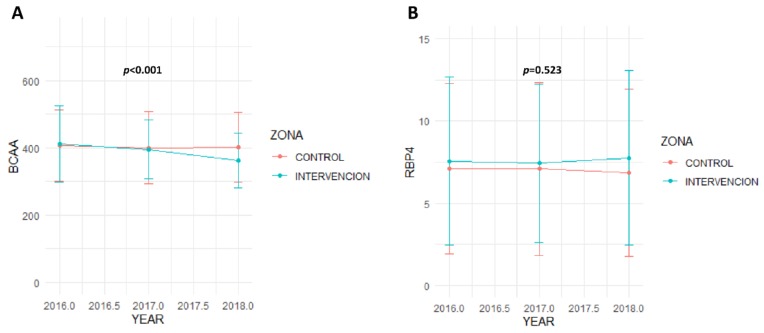
BCAA and RBP4 variation over time, depending on the group. BCAA: branched amino acid. RBP4: retinol-binding protein 4. The *p*-value, which compares BCAA variation (**A**) or RBP4 variation (**B**) over time depending on the group, was calculated by ANOVA test.

Especially interesting is the significant decline in BCAA concentration in those subjects in the intervention group compared with the control group throughout the follow-up (*p* < 0.001 between both groups). BCAA concentration change followed a linear decrease in the intervention group, getting lower concentrations at the end of the follow-up. However, RBP4 variation did not show significant variation in any group. Baseline RBP4 levels were slightly higher in the intervention group than in the control group, although no significant differences were observed between groups. These differences remained throughout the study and even increased a little at the end of the study, but always remained insignificant (*p* = 0.523, Figure 4).

**Figure 5:**
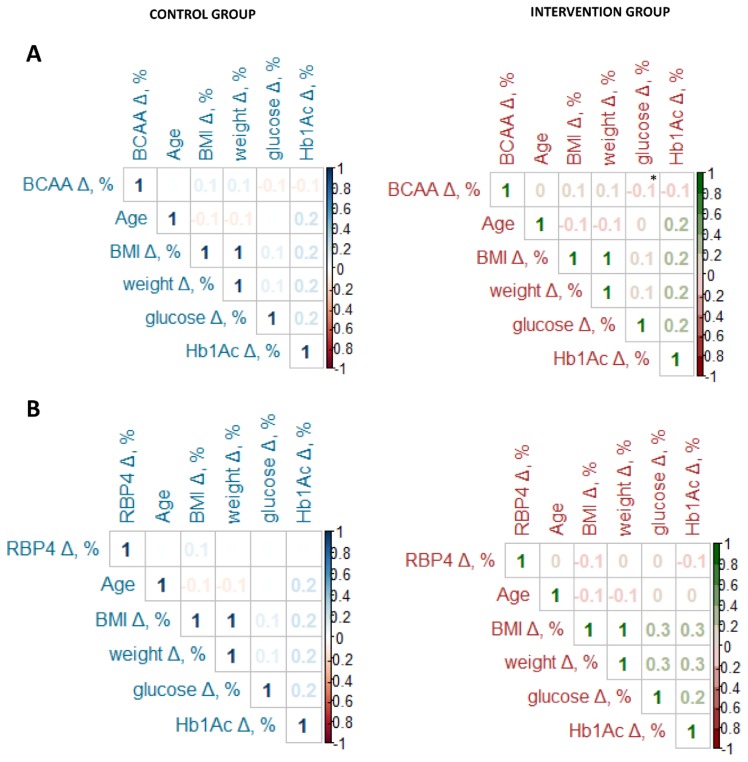
Correlation between BCAA (**A**) or RBP4 (**B**) variation with diabetes markers depending on the group. BCAA: branched amino acid. RBP4: retinol-binding protein 4. BMI: body mass index. HbA1c: glycated hemoglobin. Δ, %: variation expressed as percentage. The variation was calculated comparing the concentration at Follow-up 2 (2018) to baseline levels (2016). Correlation analyzed was performed by Pearson’s tests. * Denotes significant difference with *p* < 0.05.

Due to the marked decrease in BCAA concentrations in the intervention group, we studied the relation between BCAA and RBP4 variations with anthropometrics, glucose metabolism, and lifestyle parameters throughout the study. Only physical activity had any relationship with BCAA and RBP4 variations, showing that participants who increased their physical activity had a higher drop of BCAA and RBP4 concentrations (*p* < 0.001, Table 4). When we analyzed the correlation between BCAA and RBP4 variation with diabetes markers depending on the group the subjects were allocated to, the glucose variation showed a significant relationship with BCAA variation only in the intervention group. Participants who had higher glucose decreases had the greatest BCAA rise (r = −0.10, *p* = 0.025). Nevertheless, regression analysis continued to show an association between glucose variation and BCAA variation throughout the follow-up study (3.731 × 10^−2^ (−0.1967 to 0.2714), *p* = 0.754). BCAA variation did not show any association with any glucose metabolism parameter in the control group (Figure 5A). Neither glycemic parameter change showed a significant relationship with the RBP4 variation in either the intervention or control groups (Figure 5B).

Finally, due to the close relationship between BCAA and RBP4 levels and body weight [22,23,24], we studied RBP4 and BCAA variations according to bodyweight percentage change. We considered it a clinically significant weight loss when 5% of bodyweight reduction, according to ADA guidelines recommendations in overweight and obesity management in patients with prediabetes and T2D [25], was achieved. BCAA variation showed a positive relationship with body weight change. These effects were observed in both groups, although a much greater decrease was observed in subjects within the intervention group. However, the wide standard deviation should be taken into account when interpreting the findings (Table 5).

## 4. Discussion

The main findings of the study include (1) BCAA concentrations directly correlated to FINDRISC score and glucose impairment (both glucose, HbA1c, insulin, and HOMA–IR) at baseline in subjects with a high-risk of developing T2D; (2) BCAA levels decreased across the study in those participants receiving the school- and community-based intervention; (3) the improvement in BCAA concentration in these subjects occurred regardless of weight loss, although a higher decrease of its levels was observed in subjects reaching higher weight loss; (4) RBP4 was not significantly associated to glucose metabolism and body weight at baseline, and its concentration did not significantly vary across the study in any group.

BCAA has increasingly been studied as playing a role in diabetes, and previous research has demonstrated that higher concentrations of these amino acids are related to insulin resistance and are predictive of T2D developing in nondiabetic subjects [6,7,9,26].

Our results are in accordance with previous research by finding that baseline BCAA concentrations are directly correlated to glucose, HbA1c, and insulin resistance, assessed by HOMA–IR, in subjects with a high risk of developing T2D. The association was statistically significant in both the participants with prediabetes, T2D, and normoglycemic status. Notably, our results revealed that the FINDRISC score was directly associated with BCAA concentration at baseline. Those participants who had a higher score showed greater levels of these amino acids at the beginning of the study. Thus, our findings point out that this questionnaire would not just be a good predictor for T2D incidence in the next 10 years but would reflect the early stages of disease pathogenesis.

In previous literature, whether lifestyle intervention could modify BCAA concentrations has barely been explored, and heterogeneous results have been revealed. In the PREDIMED trial, it was demonstrated that a Mediterranean diet rich in extra-virgin olive oil significantly decreased the levels of BCAA and attenuated the positive association between plasma BCAA concentration and T2D incidence [27]. Zheng Y et al. reported significant reductions in BCAA levels both in the POUNDS LOST (Preventing Overweight Using Novel Dietary Strategies Trial) and the DIRECT (Dietary Intervention Randomized Controlled Trial) studies [28]. However, a recent study exploring the effect of a low-calorie diet with standard protein content (0.8 g/kg/day) or a high-protein content (1.2 g/kg/day) showed no statistically significant changes in BCAA concentrations despite a −6.2% and −7.2% weight loss after interventions were reached in each diet group, respectively [29]. Even though other research previously described greater levels of BCAA in subjects who were overweight or obese, the association between weight loss and BCAA change differs among studies [30,31,32]. In both the POUNDS LOST and in the DIRECT trials, weight loss was directly related to the reduction of BCAA by describing a ∼0.6 μmol/L decrease in log tyrosine per kilogram of weight loss [28]. The PREDIMED study found that elevated plasma levels of BCAA were associated with higher T2D regardless of weight loss, although the association of these amino acids, concentration, and body weight change was not shown [27]. Our study is the first one describing the effect of a school- and community-based intervention, focused not on weight loss but on the improvement of dietary and physical activity habits on BCAA concentrations in individuals with a high risk of developing T2D. These small differences between the control and intervention groups throughout the follow-up could be explained, on one hand, by the study design, which did not seek the weight loss of the participants, but the improvement of their dietary habits and physical exercise. On the other hand, in this study, we included all Spanish participants who attended the three study visits by involving an 18.6% drop-out rate, which was especially high during the first year. This relatively high drop-out is quite common within community-based intervention studies, especially when developing in vulnerable populations. However, this is an important issue that would be necessary to consider when interpreting the main outcomes of the study. Our findings demonstrate that this intervention led to a significant reduction in these amino acid levels despite the participants scarcely varying their body weight. We did not find a significant correlation between weight loss and change in BCAA levels across the study, and those participants receiving intervention showed statistically significant reductions in these metabolites regardless of weight loss. However, it is important to note that the subjects who reached >5%-weight loss showed a higher decrease in BCAA levels than those who gained body weight or subjects who had <5%-weight loss, although no significant differences were founded. Our results demonstrate that a decrease in BCAA concentrations can be reached with cost-effective community-based interventions focused on healthy lifestyles beyond weight loss.

The mechanisms linking BCAA concentrations and glucose metabolism and T2D are not fully understood, and it is rarely even discussed if elevated concentrations of these amino acids could be a cause or a consequence of insulin resistance. High levels of insulin have been demonstrated to cause impaired function of branched-chain aminotransferase (BCAT) and branched-chain keto acid dehydrogenase (BCKDH), which are enzymes with a key role in BCAA metabolism, and subjects with T2D show decreased skeletal muscle BCAT and BCKDH expression [33,34]. This impairment leads to the accumulation of branched-chain keto acids and metabolites such as diacylglycerol and ceramide, which potentially contribute to the development of further insulin resistance. Another hypothesis includes the mammalian target of rapamycin complex 1 (mTORC1), which is activated by BCAA but also by insulin and glucose via cellular ATP availability. BCAA overload may cause insulin resistance by the activation of mTOR signaling, resulting in persistent IRS-1 phosphorylation by mTORC1 and inhibition of insulin signaling [10,35]. A recent study has demonstrated that brown adipose tissue actively utilizes BCAA in the mitochondria for thermogenesis and promotes systemic BCAA clearance [12]. Our study findings, like in the PREDIMED trial, point out that BCAA concentrations decrease after lifestyle intervention regardless of body weight. So distinct, yet non-mutually exclusive mechanisms would be expected to be responsible for the physiological effects described for these amino acids.

RBP4 is an adipokine that has been implicated in the pathophysiology of insulin resistance through immunity, inflammatory, and GLUT4 regulation mechanisms in adipose and vascular tissues [14,15,36,37,38]. Several studies have demonstrated that a low-calorie diet considerably decreases RBP4 levels and that effect is dependent on the amount of weight loss [16,39]. A few studies have found that lifestyle intervention causes a decrease in RBP4 concentrations regardless of weight loss [40,41]. In our study, we did not find a significant variation of RBP4 levels after study intervention, and neither an association between RBP4 nor body weight was shown. The Feel4Diabetes trial intervention was not focused on weight loss but on the improvement of dietary and physical habits. The participants of our trial reached a ~1% of weight loss, which would be insufficient to lead to RBP4 changes according to previous researches. We did not find a significant association between RBP4 concentration and glucose metabolism parameters at baseline, and no association between glucose and HbA1c changes and RBP4 variation across the study was observed. The participants of our study were mostly normoglycemic, which could have played an essential role in the lack of association between RBP4 and glucose homeostasis, which is mainly described in subjects with glucose impairment.

Our study has several limitations worth mentioning. Firstly, dietary assessment methodology did not allow us to quantify both energy and different nutrients intake that would be of interest to know if some specific dietary characteristics could impact in the change of both in BCAA and RBP4 concentrations. On the other hand, the dropout rate of the study was relatively high, which could limit the findings of the study. However, this issue is common in long-term epidemiological and interventional studies, especially those developed in vulnerable groups of subjects.

In conclusion, our findings reveal that BCAA levels were directly related to the FINDRISC score and glucose impairment in subjects with a high-risk of developing T2D. In addition, our study shows that school- and community-based interventions focused on lifestyle improvement could lead to an improvement in BCAA concentrations regardless of weight loss. Therefore, we hypothesize that the decrease of BCAA levels could be explained by the combination of lifestyle changes and a small decrease in body weight. We did not find a significant association between RBP4 concentrations and glucose metabolism or body weight in study participants at baseline and, moreover, the intervention did not significantly influence RBP4 levels. According to our results, we believe that two main issues should be highlighted. Firstly, FINDRISC should be predictive of a high risk of developing T2D, including early stages of disease pathogenesis. Secondly, a community-based intervention has demonstrated to lead to a decrease in BCAA concentrations, of which high concentrations have been previously related to TDM2 development. Therefore, to prevent or delay the onset of T2D by identifying those individuals at high risk and finding optimal approaches to reach this goal should be a priority for both the political and scientific communities.

## Figures and Tables

**Table 1:** Baseline biochemical and anthropometric characteristics of all Spanish subjects who have completed the Feel4Diabetes study.

		Control Group (N = 115)	Intervention Group (N = 151)	*p*
**Age, years**		42.5 ± 5.13	42.0 ± 5.26	0.483
**Men, n (%)**		39 (33.9%)	51 (33.5%)	0.682
**Baseline body weight, kg**		78.7 ± 17.5	80.1 ± 17.1	0.508
**Baseline BMI, kg/m^2^**		28.5 ± 5.75	28.9 ± 5.20	0.502
**Baseline waist, cm**		96.4 ± 13.3	98.1 ± 12.8	0.293
**Baseline total cholesterol, mg/dL**		202 ± 34.6	198 ± 34.6	0.326
**Baseline LDL cholesterol, mg/dL**		127 ± 29.7	124 ± 29.4	0.365
**Baseline triglycerides, mg/dL**		88.0 (31.0–118)	80.0 (63.0–119)	0.499
**Baseline HDL cholesterol, mg/dL**		54.6 ± 12.7	53.7 ± 11.1	0.550
**Baseline apolipoprotein A, mg/dL**		169 ± 32.5	167 ± 24.6	0.642
**Baseline apolipoprotein B, mg/dL**		96.9 ± 23.9	95.9 ± 25.9	0.583
**Baseline glucose, mg/dL**		92.4 ± 18.1	91.9 ± 15.4	0.621
**Baseline insulin, UI**		6.04 ± 3.68	7.21 ± 4.81	0.027
**Baseline HbA1c, %**		5.53 ± 0.75	5.51 ± 0.44	0.569
**Baseline HOMA-IR**		1.35 ± 0.85	1.68 ± 1.30	0.045
**Baseline GGT, U/L**		27.3± 20.5	27.1 ± 26.1	0.722
**Baseline GOT, U/L**		20.8± 7.63	21.3 ± 6.60	0.211
**Baseline GPT, U/L**		22.0 ± 14.9	20.3 ± 11.5	0.737
**Baseline DM, n (%)**	**Prediabetes ^1^**	10 (8.69%)	12 (7.95%)	0.776
	**Diabetes ^2^**	4 (3.48%)	8 (5.26%)	
**Baseline score FINDRISK**		12.0 (10.0–13.0)	11.0 (9.50–13.0)	0.224
**Baseline BCAA, nmol/nL**		406 ± 105	411 ± 113	0.745
**Baseline RBP4, ng/ml**		7.09 ± 5.18	7.57 ± 5.12	0.327
**Physical activity, steps/day**		6071 ± 3387	7082 ± 2756	0.364

BMI: body mass index. LDL: low density lipoprotein. HDL: high density lipoprotein. HOMA: homeostatic model assessment. GGT: gamma-glutamyl transferase. GOT: glutamic oxaloacetic transaminase. GPT: glutamic-pyruvate-transaminase. HbA1c: glycated hemoglobin. BCAA: branched amino acid. RBP4: retinol-binding protein 4. Physical activity was measured as the total steps per day. Quantitative variables are expressed as means ± standard deviations, except for variables not following normal distribution that are expressed as medians (interquartile ranges). Qualitative variables are expressed as percentages. The *p*-value was calculated by *t*-test or U Mann–Whitney and chi-square, as appropriate. ^1^ We defined prediabetes: subjects with baseline glucose levels >100 and <126 mg/dL and HbA1c between >5.7% and <6.5%. ^2^ We defined diabetes: subjects with baseline glucose levels ≥126 mg/dL and/or HbA1c ≥6.5%.

**Table 2:** Baseline biochemical and anthropometric characteristics and baseline BCAAs and RBP4 concentrations according to impaired glycemic metabolism.

	Diabetic Subjects ^1^ (N = 12)	Prediabetic Subjects ^2^ (N = 22)	Normoglycemic Subjects (N = 232)	*p*
**Age, years**	43.5 ± 3.02	43.8 ± 5.23	42.0 ± 5.27	0.237
**Men, n (%)**	7 (58.3%)	13 (59.1%)	70 (30.2%)	0.004
**Baseline body weight, kg**	89.9 ± 20.8	83.6 ± 15.2	78.6 ± 17.1	0.043
**Baseline BMI, kg/m^2^**	32.1 ± 7.02	30.0 ± 4.37	28.5 ± 5.39	0.045
**Baseline waist, cm**	106 ± 19.4	100 ± 8.62	96.8 ± 12.9	0.062
**Baseline total cholesterol, mg/dL**	196 ± 41.3	210 ± 35.5	199 ± 34.1	0.357
**Baseline LDL cholesterol, mg/dL**	113 ± 32.6	133 ± 28.8	125 ± 29.4	0.183
**Baseline triglycerides, mg/dL**	126 (109–187)	87.5 (70.5–169)	81.0 (62.0–111)	0.020
**Baseline HDL cholesterol, mg/dL**	49.4 ± 10.9	53.5 ± 9.62	54.4 ± 12.0	0.356
**Baseline glucose, mg/dL**	146 ± 43.1	106 ± 5.19	88.1 ± 6.44	<0.001
**Baseline insulin, UI**	10.1 ± 7.09	7.49 ± 3.90	6.52 ± 4.29	0.104
**Baseline HbA1c, %**	7.24 ± 1.76	5.70 ±0.24	5.41 ± 0.29	<0.001
**Baseline HOMA-IR**	3.15 ± 2.48	2.00 ± 1.16	1.44 ± 1.02	0.001
**Baseline BCAA, nmol/nL**	486 ± 114	435 ± 69.3	403 ± 111	0.018
**Baseline RBP4, ng/ml**	7.92 ± 3.55	8.24 ± 3.99	7.25 ± 5.31	0.192
**Physical activity, steps/day**	4291 ± 1293	6990 ± 3114	6930 ± 2947	0.466

BMI: body mass index. LDL: Low density lipoprotein. HDL: high density lipoprotein. HOMA: homeostatic model assessment. HbA1c: glycated hemoglobin. BCAA: branched amino acid. RBP4: retinol-binding protein 4. Physical activity was measured as the total steps per day. Quantitative variables are expressed as means ± standard deviations; qualitative variables are expressed as percentages. The *p*-value was calculated by ANOVA test or Kruskall–Wallis and chi-square, as appropriate. ^1^ We defined prediabetes: subjects with baseline glucose levels >100 and <126 mg/dL and HbA1c between >5.7% and <6.5%. ^2^ We defined diabetes: subjects with baseline glucose levels ≥126 mg/dL and/or HbA1c ≥6.5%.

**Table 3:** Evolution of biochemical and anthropometric characteristics of all Spanish control and intervention subjects who have completed the Feel4Diabetes study.

	Control Group (N = 115)			Intervention Group (N = 151)			Overall *p*
	2016 (N = 115)	2017 (N = 115)	2018 (N = 115)	2016 (N = 151)	2017 (N = 151)	2018 (N = 151)	
**Men, n (%)**	39 (33.9%)	39 (33.9%)	39 (33.9%)	51 (33.5%)	51 (33.5%)	51 (33.5%)	0.682
**Body weight, kg**	78.7 ± 17.5	77.5 ± 16.0 *	77.9 ± 16.4	80.1 ± 17.1	79.2 ± 16.7 *	79.5 ± 16.8	0.638
**BMI, kg/m^2^**	28.5 ± 5.75	28.3 ± 5.71	28.4 ± 5.77	28.9 ± 5.20	28.3 ± 5.23 *	28.5 ± 5.21	0.703
**Total cholesterol, mg/dL**	202 ± 34.6	198 ± 35.1	196 ± 37.3	198 ± 34.6	196 ± 34.2	199 ± 34.1	0.068
**LDL cholesterol, mg/dL**	127 ± 29.7	122 ± 30.8	124 ± 34.5	124 ± 29.4	120 ± 27.8	124 ± 28.2	0.344
**Triglycerides, mg/dL**	88.0 (31.0–118)	78.0 (61.0–111) *	81.0 (57.5–119) **	80.0 (63.0–119)	79.0 (60.5–116)	84.0 (63.0–113)	0.542
**HDL cholesterol, mg/dL**	54.6 ± 12.7	56.4 ± 13.0*	54.6 ± 11.4	53.7 ± 11.1	56.4 ± 11.5*	55.1 ± 11.8 **	0.089
**Apolipoprotein A, mg/dL**	169 ± 32.5	167 ± 28.6	144 ± 21.2 **	167 ± 24.6	172 ± 25.8*	145 ± 20.0 **	0.334
**Apolipoprotein B, mg/dL**	96.9 ± 23.9	95.8 ± 21.9	88.1 ± 24.0 **	95.9 ± 25.9	94.9 ± 24.8	88.5 ± 21.1 **	0.264
**Glucose, mg/dL**	92.4 ± 18.1	95.6 ± 33.8 *	92.6 ± 18.0	91.9 ± 15.4	93.0 ± 15.1	90.6 ± 11.7	0.417
**HbA1c, %**	5.53 ± 0.75	5.46 ± 0.81 *	5.36 ± 0.33 **	5.51 ± 0.44	5.40 ± 0.53*	5.36 ± 0.36 **	0.011
**GGT, U/L**	27.3± 20.5	27.0 ± 21.1	28.1 ± 26.4	27.1 ± 26.1	27.5 ± 25.0	26.3 ± 21.7	0.271
**GOT, U/L**	20.8± 7.63	21.8 ± 8.81	21.6 ± 8.81	21.3 ± 6.60	22.7 ± 9.45	21.4 ± 4.94	0.489
**GPT, U/L**	22.0 ± 14.9	22.4 ± 17.5	22.4 ± 17.5	20.3 ± 11.5	22.3 ± 15.2	22.3 ± 15.2	0.332
**BCAA, nmol/nL**	406 ± 105	400 ± 108	402 ± 103	411 ± 113	395 ± 86.8*	363 ± 80.5 **	<0.001
**RBP4, ng/mL**	7.09 ± 5.18	7.09 ±5.27	6.87 ± 5.09	7.57 ± 5.12	7.42 ± 4.83	7.76 ± 5.31	0.523
**Physical activity, steps/day**	4637 ± 2885	5544 ± 3433	6127 ± 3808	5601 ± 1834	5851 ± 2349	7208 ± 2543	0.273

BMI: body mass index. LDL: low density lipoprotein. HDL: high density lipoprotein. HOMA: homeostatic model assessment. GGT: gamma-glutamyl transferase. GOT: glutamic oxaloacetic transaminase. GPT: glutamic-pyruvate-transaminase. HbA1c: glycated hemoglobin. BCAA: branched chain amino acid. RBP4: retinol-binding protein 4. Physical activity was measured as the total steps per day. Quantitative variables are expressed as means ± standard deviations except for variables not following normal distribution that are expressed as medians (interquartile ranges). Overall *p* was calculated by mixed lineal model comparing the variation during the time in control vs. intervention group. * Denotes significant difference between baseline year (2016) and the first year to intervention (2017) in each group calculated by *t*-test or U Mann–Whitney as appropriate. ** Denotes significant difference between baseline year (2016) and the second year to intervention (2018) in each group calculated by *t*-test or U Mann–Whitney as appropriate.

**Table 4:** Relationship between BCAA variation and RBP4 variation with variation of anthropometric, diabetic markers, dietary markers, and physical exercise parameters throughout the Feel4Diabetes study (2016–2018).

	BCAA Variation				*p*	*p^1^*	RBP4 Variation				*p*	*p^1^*
	Q1 (N = 67)	Q2 (N = 66)	Q3 (N = 65)	Q4 (N = 68)			Q1 (N = 66)	Q2 (N = 66)	Q3 (N = 66)	Q4 (N = 68)		
**BCAA variation, %**	−28.3 ± 9.03	−10.8 ± 3.79	−0.42 ± 3.16	18.8 ± 12.4	<0.001	<0.001	−2.13 ± 20.4	−4.17 ± 18.4	−9.11 ± 18.2	−5.35 ± 18.5	0.194	0.343
**RBP4 variation, %**	22.8 ± 80.7	8.98 ± 55.7	15.4 ± 75.4	13.2 ± 60.6	0.706	0.440	−41.5 ± 14.6	−11.0 ± 6.62	16.7 ± 9.33	96.2 ± 90.0	<0.001	<0.001
**BMI variation, %**	−0.79 ± 7.43	−1.73 ± 8.37	0.34 ± 6.33	0.58 ± 5.33	0.201	0.224	−0.60 ± 6.54	−0.50 ± 6.11	−0.36 ± 7.56	−0.16 ± 7.68	0.987	0.731
**Body weight variation, %**	−0.67 ± 7.58	−1.37 ± 8.29	0.56 ± 5.12	0.63 ± 5.74	0.260	0.263	−0.27 ± 6.68	−0.33 ± 6.15	−0.13 ± 7.71	−0.11 ± 6.82	0.997	0.894
**Glucose variation, %**	0.25 ± 9.46	−1.43 ± 11.2	0.20 ± 9.15	0.71 ± 9.86	0.624	0.785	−0.63 ± 8.26	0.70 ± 9.98	1.11 ± 10.2	−1.38 ± 11.1	0.437	0.654
**Hb1Ac variation, %**	−1.33 ± 5.62	−2.74 ± 3.41	−1.65 ± 3.84	−1.84 ± 3.56	0.263	0.537	−1.79 ± 3.78	−1.68 ± 2.98	−1.41 ± 5.77	−2.63 ± 3.87	0.379	0.209
**Vegetables variation consumption (g/week), %**	55.2 ± 179	52.5 ± 182	35.7 ± 166	55.3 ± 176	0.919	0.995	45.4 ± 172	67.5 ± 227	37.7 ± 160	47.2 ± 127	0.825	0.951
**Legume variation consumption (g/week), %**	49.3 ± 114	24.1 ± 105	88.6 ± 218	52.4 ± 144	0.216	0.907	46.2 ± 125	39.6 ± 149	39.0 ± 97.4	91.4 ± 216	0.292	0.227
**Read meat variation consumption (g/week), %**	2.21 ± 72.6	-20.0 ± 62.3	52.3 ± 149	4.76 ± 73.4	0.002	0.862	10.4 ± 110	10.4 ± 75.4	10.7 ± 94.4	14.6 ± 121	0.996	0.856
**Fish variation consumption (g/week), %**	14.5 ± 72.1	-2.88 ± 65.5	25.7 ± 123	39.4 ± 96.0	0.133	0.145	18.1 ± 69.2	23.0 ± 117	19.8 ± 97.8	19.7 ± 90.0	0.995	0.919
**Physical activity variation (number steps/day), %**	48.5 ± 64.9	14.9 ± 43.3	50.7 ± 65.6	−18.9 ± 45.5	0.537	0.001	58.7 ± 78.9	18.7 ± 28.9	7.44 ± 41.2	18.0 ± 59.7	0.696	<0.001

BMI: body mass index. HbA1c: glycated hemoglobin. BCAA: branched amino acid. Physical activity was measured as the total steps per day. RBP4: retinol-binding protein 4. Quantitative variables are expressed as means ± standard deviations. ^2^ *p* refers to differences among all quartiles in each study group and was calculated by ANOVA or Kruskall–Wallis, as applicable. ^3^ *p* refers to differences comparing Q1 and Q4 quartiles in each study group and was calculated by *t*-test or U Mann–Whitney, as applicable.

**Table 5:** Relationship between BCAA and RBP4 variations according to weight change experienced during the study in each group.

	Control Group			*p* ^2^	Intervention Group			*p* ^2^
	Subjects who Have Lost more than 5% of Weight throughout the F4D Study (N = 19)	Subjects who Have Varied less than 5% of Weight throughout the F4D Study (N = 78)	Subjects who Have Gained more than 5% of Weight throughout the F4D Study (N = 17)		Subjects who Have Lost more than 5% of Weight throughout the F4D Study (N = 21)	Subjects who Have Varied less than 5% of Weight throughout the F4D Study (N = 103)	Subjects who Have Gained more than 5% of Weight throughout the F4D Study (N = 24)	
**BCAA variation, %**	−1.48 ± 17.2	−0.11 ± 19.9	5.58 ± 16.8	0.493	−10.9 ± 28.6	−9.55 ± 15.3	−7.14 ± 17.3	0.768
**RBP4 variation, %**	16.8 ± 73.4	10.9 ± 54.0	9.27 ± 36.3	0.904	44.2 ± 129	13.1 ± 51.8	24.2 ± 104	0.232

BCAA: branched chain amino acid; RBP4: retinol binding protein 4. Variables are expressed as means ± standard deviations. *p*^2^ refers to differences among the three groups in each study group and was calculated by ANOVA or Kruskall–Wallis, as applicable.

## Supplementary Materials

The following are available online at https://www.mdpi.com/2073-4409/9/3/693/s1. Table S1. Baseline BCAA and RBP4 levels according to FINDRISCK items. Figure S1. Flow chart. Figure S2. BCAA concentration evolution according to sex in both groups. BCAA: branched amino acid. Quantitative variables are expressed as means ± standard deviations. The *p*^1^ compared the evolution of biochemical and anthropometric characteristics along to follow-up. The *p*^2^ compared baseline BCAA concentrations according to sex groups. The *p*-value was calculated by ANOVA or Student’s test, as appropriate. Figure S3. Correlation between baseline levels of BCAA and RBP4 with clinical parameters (N = 266). BCAA: branched amino acid. TG: triglycerides. HDLc: high density lipoprotein cholesterol. LDLc: low density lipoprotein cholesterol. GGT: gamma-glutamyl transferase. GOT: glutamic oxaloacetic transaminase. GPT: glutamic-pyruvate-transaminase. SBP: systolic blood pressure. DBP: diastolic blood pressure. RBP4: retinol-binding protein 4. Correlation analyzed was performed by Pearson’s tests. * Denotes significant difference with *p* < 0.05.
