# Supplementary material for: Effect of Lifestyle Intervention in the Concentration of Adipoquines and Branched Chain Amino Acids in Subjects with High Risk of Developing Type 2 Diabetes: Feel4Diabetes Study
Source: Cells. 2020 Mar 12;9(3):693. doi: 10.3390/cells9030693 (PMC7140606; doi:10.3390/cells9030693)
Supplement: Supplementary file 1 [file cells-09-00693-s001.zip › supple-revised/supple-table.pdf]

Supplemental table 1. Baseline BCAA and RBP4 levels according to FINDRISCK items.[1]

FINDRISC item		Baseline BCAA (nmol/nL)	P^2	Baseline RBP4 (ng/mL)	P^3
Age (years)	<45 (N=172)	401 ±108	0.243	7.33 ±5.11	0.483
	45-54 (N=92)	423 ±113		7.48 ±5.25	
	55-64 (N=1)	467		2.07	
Self-reported BMI (kg/m²)	<25 (N=93)	374 ±72.2	**<0.001**	7.29 ±4.26	0.260
	25-30 (N=93)	423 ±104		7.73 ±5.23	
	>30 (N=79)	435 ±140		6.99 ±6.01	
Self-reported waist circumference: women/men (cm)	<80/<94 (N=24)	375 ±74.5	0.244	9.61 ±5.82	0.093
	80-88/94-102 (N=88)	407 ±82.3		7.04 ±4.64	
	>88/>102(N=153)	416 ±127		7.19 ±5.27	
Physical activity (min)	>30 min/day (N=143)	413 ±116	0.573	7.83 ±5.36	0.101
	<30 min/day (N=122)	405 ±103		6.80 ±4.86	
Fruit, vegetables and berries consumption	Everyday(N=193)	404 ±98.8	0.238	7.19 ±4.70	0.929
	Not Everyday (N=72)	422 ±135		7.81 ±6.22	
Blood pressure medication	No (N=237)	406 ±101	0.235	6.93 ±4.91	**<0.001**
	Yes (N=28)	433 ±168		11.1 ±5.78	
High glucose levels ever	No (N=179)	417 ±110	0.120	7.35 ±5.41	0.550
	Yes (N=86)	394 ±108		7.38 ±4.60	
T2D family history	No (N=52)	404 ±102	0.730	7.98 ±5.97	0.800
	Yes, first relative (N=137)	406 ±82.2		7.18 ±4.41	
	Yes, second relative (N=76)	418 ±152		7.27 ±5.80	

[1]Variables are expressed as means ± standard deviations. [2]*p* refers to differences in baseline BCAA levels according to different categories for each FINDRISC item calculated by t-test, ANOVA, Kruskal-Wallis test or U Mann Whitney test, as applicable. [3]*p* refers to differences in baseline RBP4 levels according to different categories for each FINDRISC item calculated by t-test, ANOVA, Kruskal-Wallis test or U Mann Whitney test, as applicable.
