# Supplementary material for: Effect of Lifestyle Intervention in the Concentration of Adipoquines and Branched Chain Amino Acids in Subjects with High Risk of Developing Type 2 Diabetes: Feel4Diabetes Study
Source: Cells. 2020 Mar 12;9(3):693. doi: 10.3390/cells9030693 (PMC7140606; doi:10.3390/cells9030693)

Supplemental Figure 2. BCAA concentration evolution according to sex in both group

Intervention group (N=151)

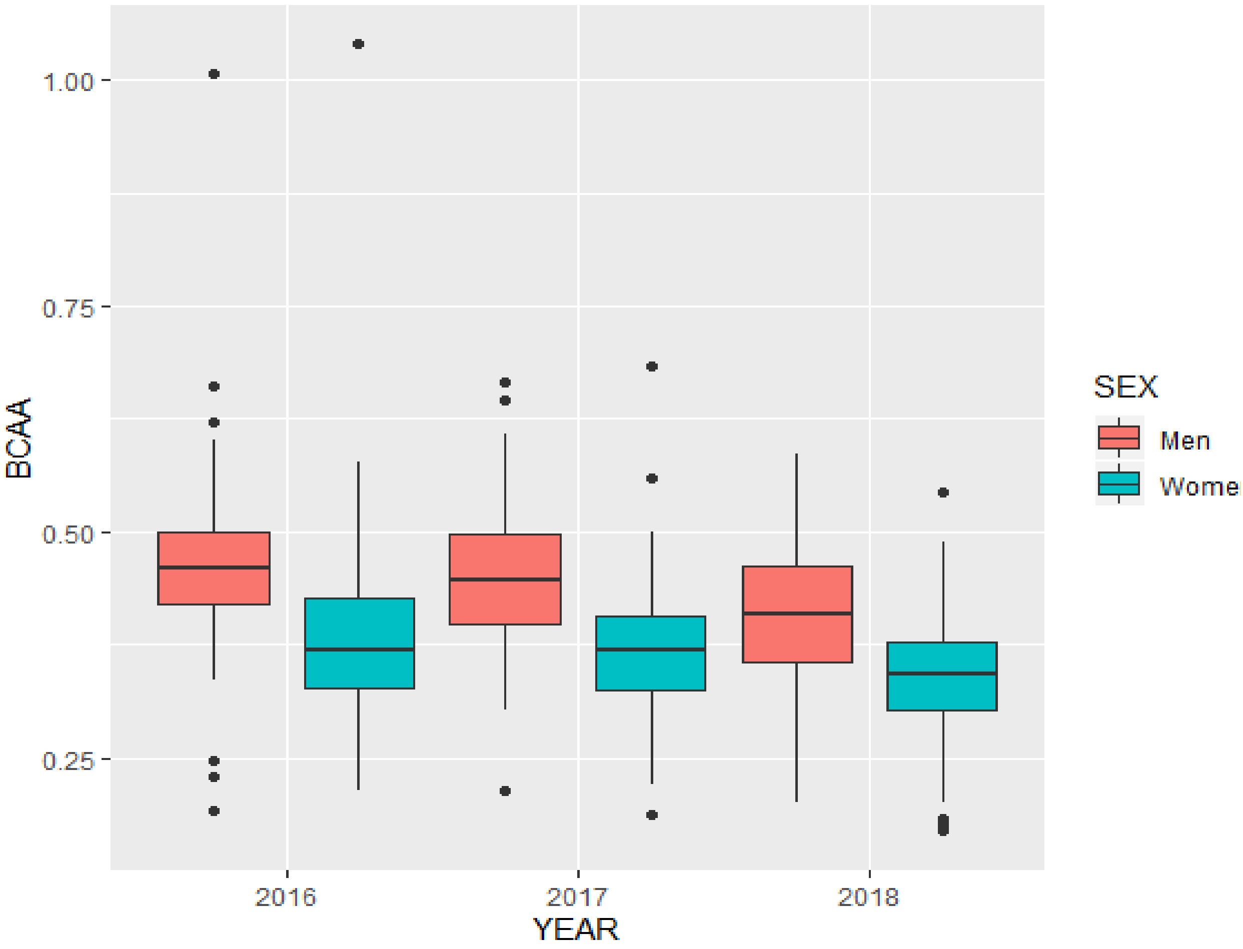

	2016 (N=151)	2017 (N=151)	2018 (N=151)	p[1]	p[2]
**Men BCAA concentration, nmole/nL (N=51)**	465 ± 118.5	449 ± 89.0	411 ± 79.8	<0.001	<0.001
**Women BCAA concentration, nmole/nL (N=100)**	383 ± 100	368 ± 72.1	338 ± 69.5	<0.001	

Control group (N=115)

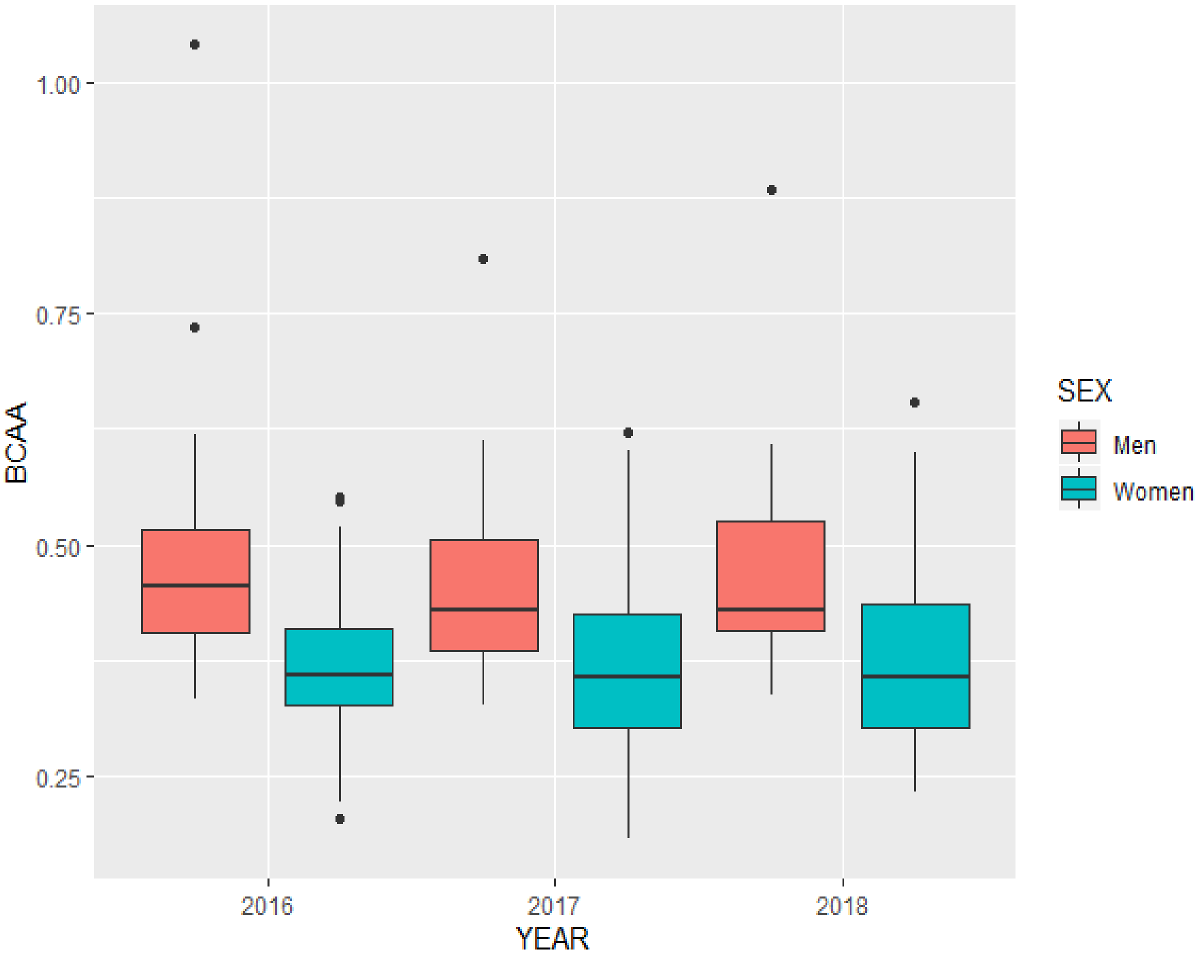

	2016 (N=151)	2017 (N=151)	2018 (N=151)	p[1]	p[2]
**Men BCAA concentration, nmole/nL (N=39)**	479 ±123	454 ± 99.2	464 ± 104	0.293	<0.001
**Women BCAA concentration, nmole/nL (N=76)**	369 ± 71.6	374 ± 103	370 ± 87.0	0.870	

Supplemental Figure 3. Correlation between Baseline levels of BCAA and RBP4 with clinical parameters (N=266)

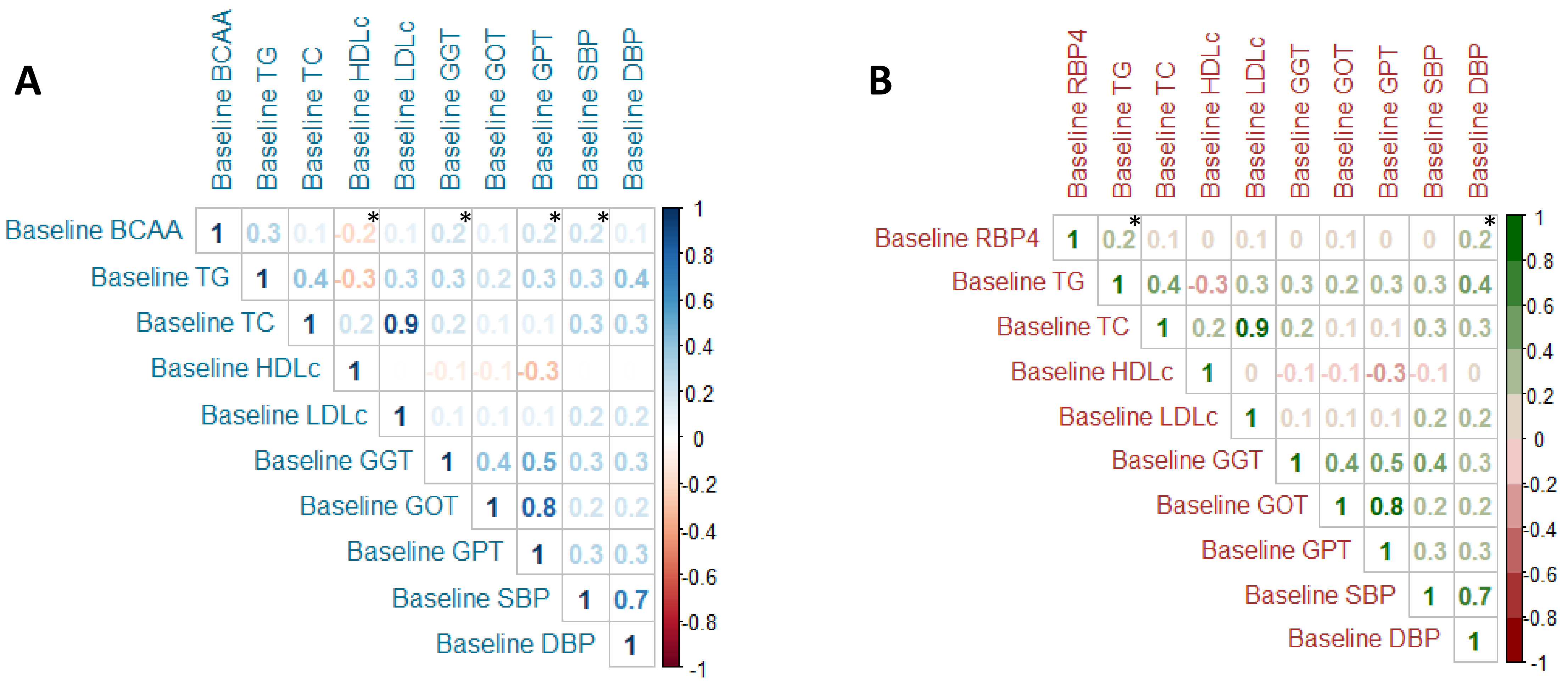

Supplement: Supplementary file 1 [file cells-09-00693-s001.zip › supple-revised/Supplemental Figures.pdf]
